# Supplementary material for: Defect-Induced Gas-Sensing Properties of a Flexible SnS Sensor under UV Illumination at Room Temperature
Source: Sensors (Basel). 2020 Oct 7;20(19):5701. doi: 10.3390/s20195701 (PMC7583859; doi:10.3390/s20195701)
Supplement: Supplementary file 1 [file sensors-20-05701-s001.pdf]

sensors

MDPI

Supplementary Materials

Defect-Induced Gas-Sensing Properties of a Flexible SnS Sensor under UV Illumination at Room Temperature

Nguyen Manh Hung [1,2], Chuong V. Nguyen [2], Vinaya Kumar Arepalli [3], Jeha Kim [3], Nguyen Duc Chinh [1], Tien Dai Nguyen [4,5], Dong-Bum Seo [1], Eui-Tae Kim [1], Chunjoong Kim [1,*] and Dojin Kim [1,*]

[1] Department of Materials Science and Engineering, Chungnam National University, Daejeon 34134, Republic of Korea; hungnm@lqdtu.edu.vn (N.M.H.); chinhnd@cnu.ac.kr (N.D.C.); Sdb987@cnu.ac.kr (D.B.S.); etkim@cnu.ac.kr (E.T.K.)
[2] Department of Materials Science and Engineering, Le Quy Don Technical University, Hanoi 100000, Vietnam; Chuong.vnguyen@lqdtu.edu.vn
[3] Department of Energy Convergence Engineering, Cheongju University, Cheongju 28503, South Korea; vinaykumararepalli@gmail.com (V.K.A.); jeha@cju.ac.kr (J.K.)
[4] Institute of Theoretical and Applied Research, Duy Tan University, Hanoi 100000, Vietnam; nguyentiendai@duytan.edu.vn
[5] Faculty of Natural Sciences, Duy Tan University, Da Nang 550000, Vietnam
* Correspondence: ckim0218@cnu.ac.kr (C.K.); dojin@cnu.ac.kr (D.K.)

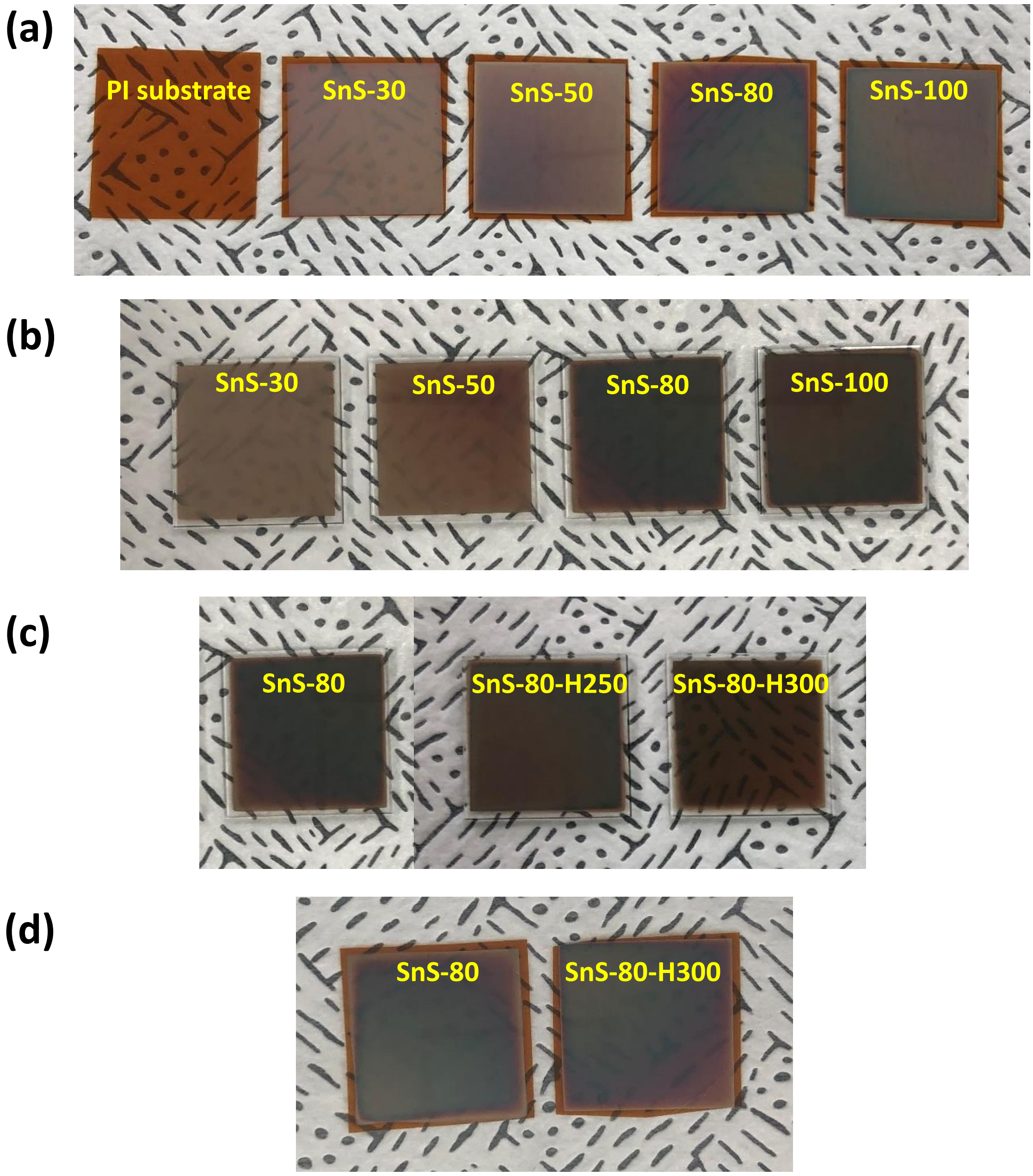

Figure S1. As-deposition SnS thin films with various thicknesses sputtered onto (**a**) polyimide substrates and (**b**) glass substrates. (**c**) SnS-80 thin films annealed at different temperatures (250 and 300 °C) compared to as-deposition SnS thin film. (**d**) SnS-80-H300 thin film on PI substrate shows no deformation after annealing at 300 °C in comparison to as-deposition SnS-80 thin film under observation by the naked eye.

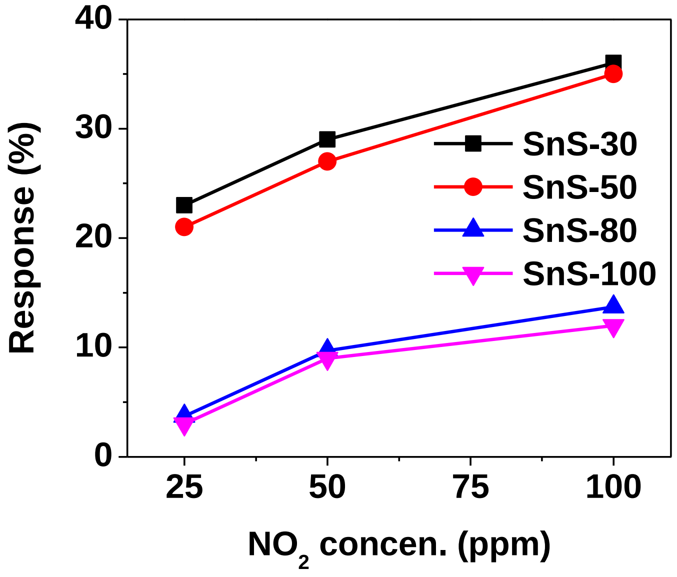

Figure S2. Summaries of the response of as-deposition SnS thin film sensors toward NO2 at RT under UV illumination (derived from Figure 7d).

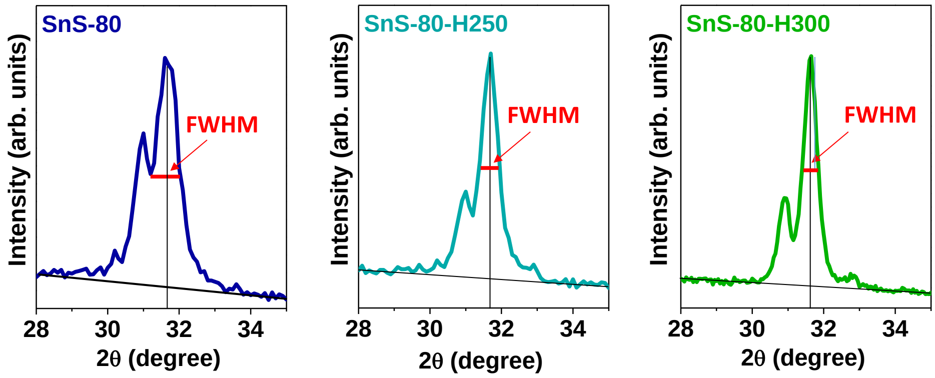

Figure S3. Enlarged XRD profiles of (004) diffraction peaks of SnS-80, SnS-80-H250, and SnS-80-H300.

The size of particles can be calculated using the Debye–Sheerer fomular:

$$d = \frac{k.\lambda}{\beta.cos\theta} \qquad (1)$$

Here, k is a constant and gets a value of 0.94.

λ is the X-ray wavelength (λ = 0.15418 nm for $Cu_{K\alpha}$ radiation).

β is the full width at half maximum or FWHM (rad).

θ is the difraction degree (degree).

The results calculated from Equation (1) are summaried in the following Table S1:

Table S1. The particle sizes of various SnS thin-films annealed at different temperatures.

Samples	FWHM [degree]	FWHM [rad]	d (nm)
SnS-80	0.840	0.0147	10.2
SnS-80-H250	0.495	0.0086	17.5
SnS-80-H300	0.48	0,0083	18.2
